# Comparative Oncology of Companion Animals

**DOI:** 10.3390/vetsci13090922

**Published:** 2026-09-08

**Authors:** Ana I. Faustino-Rocha, Paula A. Oliveira, Rui M. Gil da Costa

**Affiliations:** 1 Centre for the Research and Technology of Agro-Environmental and Biological Sciences (CITAB), University of Trás-os-Montes and Alto Douro (UTAD), 5000-801 Vila Real, Portugal; anafaustino@uevora.pt (A.I.F.-R.); pamo@utad.pt (P.A.O.); 2 Institute for Innovation, Capacity Building and Sustainability of Agri-Food Production (Inov4Agro), University of Trás-os-Montes and Alto Douro (UTAD), 5000-801 Vila Real, Portugal; 3 Comprehensive Health Research Center, Department of Zootechnics, University of Évora, 7000-812 Évora, Portugal; 4 Department of Pathology, State University of Maranhão (UEMA), São Luís 65055-310, Brazil; 5 Molecular Oncology and Viral Pathology Group, Research Center of IPO Porto (CI-IPOP)/RISE@CI-IPOP (Health Research Network), Portuguese Oncology Institute of Porto (IPO Porto)/Pathology and Laboratory Medicine Department/Clinical Pathology/Porto Comprehensive Cancer Center Raquel Seruca (Porto. CCC), 4200-072 Porto, Portugal; 6 Laboratory for Process Engineering, Environment, Biotechnology and Energy (LEPABE), Associate Laboratory in Chemical Engineering (ALiCE), Faculty of Engineering, University of Porto (FEUP), 4200-465 Porto, Portugal

Cancer constitutes a significant clinical challenge in veterinary medicine, with a high prevalence among companion animals. While cutaneous and mammary neoplasia account for a large proportion of malignant lesions in companion animals, other malignant tumors are also frequently found, for instance, in the neurological, gastrointestinal, and urological systems [1]. Despite recent advances concerning the early diagnosis of cancer and its treatment [2], it remains among the most common causes of death in companion animals. Furthermore, companion animals with naturally occurring cancers are widely utilized as a natural model for cancer research [3]. In fact, dogs and cats develop multiple types of cancer with numerous clinical, molecular, histopathological, and genetic similarities to human cancers [4]. Such similarities constitute opportunities to develop new diagnostic and prognostic markers and new therapies for human and animal diseases [3,4,5]. Despite the availability of numerous treatment approaches for cancer, advancements in the understanding of cancer mechanisms and the development of innovative treatments are still needed to improve outcomes for human and animal oncologic patients. Work on improving methods for earlier diagnosis and stratifying cancer patients according to their response to available therapies is also ongoing. Accordingly, this Special Issue, entitled “Comparative Oncology of Companion Animals”, aimed to publish original research and reviews concerning the diagnosis, treatment, and prognosis of cancer in companion animals, highlighting new advances in this field.

This Special Issue included four research articles and one review article. Two and one of these articles deal with canine and feline diseases, respectively, one examines both, and the fifth article deals with an experimental rat model of mammary cancer, which remains a major health issue for companion animals and humans. The single review article included in this Special Issue [6] addresses key aspects of the tumor microenvironment in feline mammary carcinomas. The authors focus on tumor necrosis, fibrosis, angiogenesis, adipose tissue-associated inflammation, extracellular vesicles, the epithelial–mesenchymal transition, and their prognostic implications. Overall, the review findings indicate that collagen remodeling, cancer-associated fibroblasts, regulatory T lymphocytes, and elevated serum leptin levels are associated with poor prognosis, while the presence of stromal cytotoxic T lymphocytes is associated with a more favorable outcome. The authors did not find consistent data regarding necrosis and pro-angiogenic factors, and report that research on the roles of extracellular vesicles on this type of cancer is still at a very early stage.

Schwinn et al. [7] contributed a research article on canine cutaneous mast cell tumors occurring in 849 dogs aged up to 3 years old. In this relatively young dog population, the authors reported on most affected breeds and anatomic locations, the tumor’s histological grade, and Ki67 and c-KIT immunohistochemical staining patterns. Danish–Swedish farm dogs and English Setters were over-represented and German Shepherd dogs were under-represented in this population. The authors concluded that cutaneous mast cell tumors in this young dog population share important similarities and differences compared with the general canine population, although survival data was not yet available.

Lisjak et al. [8] also reported on dogs with mast cell tumors (MCTs) but studied the fecal microbiota of a specific dog population whose tumors were treated with electrochemotherapy and IL-12 gene electrotransfer. The authors reported that dogs with MCTs showed differential relative abundance of specific bacteria compared with normal controls, although there were no differences in the dysbiosis index, alpha diversity, or beta diversity, nor between the expression levels of immunological markers such as PD-1 and granzyme B. The authors also performed analyses before and after the treatment with electrochemotherapy and IL-12 gene electrotransfer and found no differences associated with this treatment.

A research article by Juodžiukynienė [9] described the prevalence of feline and canine tumors in Lithuania. Mammary and cutaneous tumors were most commonly diagnosed. In agreement with global trends, cutaneous squamous cell carcinomas were more common in cats.

Silva et al. [10] contributed an experimental research article describing the effects of resistance training on the development of chemically induced mammary cancer in Wistar rats, a relevant model of cancer. The authors observed that exercise training thrice a week on a ladder resulted in earlier tumor onset, a slightly higher tumor incidence, and a lower mortality rate. Additional studies are required to clarify the potential role of exercise training on the risk and biopathological characteristics of mammary cancer in companion animals.

Taken together, the articles published in this Special Issue reflect the very diverse approaches to companion animal oncology, and the integration of concepts from other scientific areas. Future research in companion animal oncology should consider gray areas and incompletely solved issues, such as the biopathological role of microvesicles in the tumor microenvironment and body fluids and their potential as diagnostic and prognostic markers. The role of the microbiome as a tumor-enabling characteristic [11] is also poorly understood in companion animals and deserves further study.
